# Supplementary material for: Mutations in PCYT2 disrupt etherlipid biosynthesis and cause a complex hereditary spastic paraplegia
Source: Brain. 2019 Oct 22;142(11):3382–97. doi: 10.1093/brain/awz291 (PMC6821184; doi:10.1093/brain/awz291)
Supplement: awz291_Supplementary_Data [file awz291_supplementary_data.zip › awz291-Suppl_data/Supplementary_Data4.zip!/Supplementary information 3 - fibroblast lipidomics.zip!/Fibroblast lipidomics/Controle vs Patient2/Statistical plots and data/Heatmap sorted on p-value.pdf]

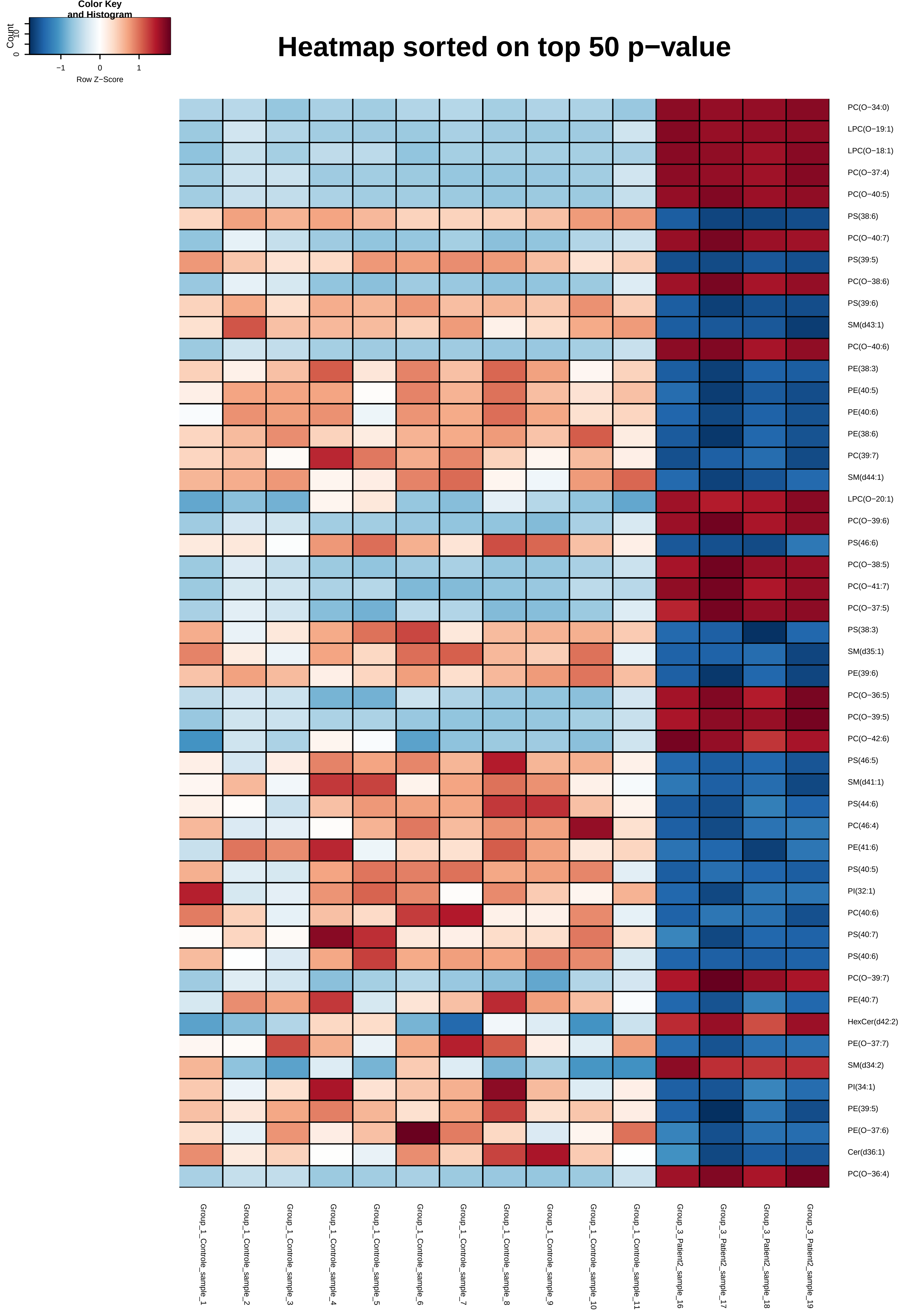

Color Key
and Histogram
Count
0
10
−1
0
1
Row Z−Score
Heatmap sorted on top 50 p−value
PC(O−34:0)
LPC(O−19:1)
LPC(O−18:1)
PC(O−37:4)
PC(O−40:5)
PS(38:6)
PC(O−40:7)
PS(39:5)
PC(O−38:6)
PS(39:6)
SM(d43:1)
PC(O−40:6)
PE(38:3)
PE(40:5)
PE(40:6)
PE(38:6)
PC(39:7)
SM(d44:1)
LPC(O−20:1)
PC(O−39:6)
PS(46:6)
PC(O−38:5)
PC(O−41:7)
PC(O−37:5)
PS(38:3)
SM(d35:1)
PE(39:6)
PC(O−36:5)
PC(O−39:5)
PC(O−42:6)
PS(46:5)
SM(d41:1)
PS(44:6)
PC(46:4)
PE(41:6)
PS(40:5)
PI(32:1)
PC(40:6)
PS(40:7)
PS(40:6)
PC(O−39:7)
PE(40:7)
HexCer(d42:2)
PE(O−37:7)
SM(d34:2)
PI(34:1)
PE(39:5)
PE(O−37:6)
Cer(d36:1)
PC(O−36:4)
Group_1_Controle_sample_1
Group_1_Controle_sample_2
Group_1_Controle_sample_3
Group_1_Controle_sample_4
Group_1_Controle_sample_5
Group_1_Controle_sample_6
Group_1_Controle_sample_7
Group_1_Controle_sample_8
Group_1_Controle_sample_9
Group_1_Controle_sample_10
Group_1_Controle_sample_11
Group_3_Patient2_sample_16
Group_3_Patient2_sample_17
Group_3_Patient2_sample_18
Group_3_Patient2_sample_19
